# Towards Near Perfect Crystals With Only Well-Characterized Imperfections

**DOI:** 10.6028/jres.106.060

**Published:** 2001-12-01

**Authors:** H. Steffen Peiser

**Affiliations:** 403 Russell Ave. Apt. 313, Gaithersburg, MD 20877-2825

**Keywords:** ARPA, crystallography, dislocations, free-radicals, melt growth, point defects, purification, solution growth, symmetry of crystals, twinning, vacancies, vapor growth

## Abstract

Throughout the past century NBS/NIST supported a great variety of research studies in crystallography where the aim was the highest attainable accuracy in measurements. While avoiding overlap with other papers in this volume, this article summarizes results from: 1) early work on crystallization as a method of purification, in contributions to sugar chemistry, and in solution growth of large crystals; 2) the NBS/ARPA Program of research on crystal growth and characterization; 3) the NBS Free-Radical Research Program; 4) the XRCD method as a direct path to relative atomic mass data and the fundamental physical constants; 5) the dynamical theory of x-ray diffraction; and 6) symmetry considerations such as are involved in the influence on crystals of mechanical stress or fields, and of point defect motion.

## 1. Introduction

During the latter half of the 19th century, the study of matter in the nearly perfect molecular disorder of the gaseous state led to the kinetic theory and thermodynamic concepts that prominently shaped progress in chemistry and physics into the next century. Since its inauguration, the National Bureau of Standards (NBS) contributed to that progress by its careful critical measurements at the highest attainable sensitivity, as is well documented in Refs. [1] and [2].

In the 20th century, the study of the nearly perfect molecular order of the solid state and its interactions with photons—that is the study of crystallography—has prominently shaped spectacular further progress in physics and chemistry. Crystallography today is at the center of all materials science and engineering, including solid-state physics, geology, metallurgy, ceramics, biophysics, and biochemistry. Thus crystallography has become a prominent key to a widening scientific understanding of our material universe, and even the life within it. NBS, now renamed the National Institute of Standards and Technology, has again contributed by its predilection to careful experimentation and critical measurements at the highest attainable sensitivity. In choosing my illustrative examples of NBS/NIST achievements in crystallography, I have avoided overlap with contributions otherwise covered in either this Special Issue or in Refs. [1] and [2], but I have given undue prominence to work with which I had some personal contact.

## 2. Crystallization

The theory of atomic scale order in crystals had long been established based on morphological and optical studies. This order could not be directly observed in the 19th century. Besides, many solids do not exhibit long- range order; they are not crystalline, but amorphous. Many crystals were long known to shatter at characteristic temperatures when cooled below their melting points. Melt growth, especially of metal crystals, had been extensively studied. Vapor growth had been observed, such as in ice crystals, since the Renaissance. Solution growth of crystals had been most widely studied, variabilities of their solubilities carefully measured, and the growth of macroscopic crystals had been demonstrated for many substances.

The resistance to being crystallized, particularly of certain sugars, was known when the problem of reliable cane sugar assay was brought to the NBS in the face of controversy between suppliers, refiners, and distributors. The Federal Government was involved by virtue of its import duties on sugar. Frederick Bates and his colleagues [3] transformed the industry, not only by perfecting the optical measuring instrument, the saccharimeter, but equally by the publication of credible measurement protocols. Expanding upon this work in typical NBS fashion, its chemical laboratory became a prominent center in the world of basic sugar chemistry that revealed fascinating details of molecular ring formulae and optically active crystal structures. This NBS work is documented, for instance, in Ref. [4].

## 3. Purification by Crystallization

It had also long been known in science and exploited by the manufacturing industry that many substances can be successfully purified from contamination by crystallization from melt and from solution. Chemists knew how to exploit the phase rule, a widely applicable development from the study of gases. They also observed that large crystals tend to be purer than small crystals of the same substance because the latter have larger ratios of surface area to crystal volume. Impurities are preferentially held at crystal grain boundaries. The selectivity of the growing crystal for incorporating molecules of only a specific type and chirality in sugars was found at NBS to depend not only on crystal phase, but also on crystal habit and temperature [4, 5].^1^

Before the implications of dislocations had been widely understood, Charles Saylor at NBS stood out among our colleagues with a deep understanding of the processes of purification by crystallization. In Ref. [6] he refined the method of microscopic measurement of refractive index by immersion practiced by mineralogists generally and currently in a critical, NIST-advocated technique for distinguishing asbestos minerals. Saylor’s group understood well how to combine crystal density with optical and chemical properties to assess the purity of crystals [7].

John Torgesen, supported by his technician, Avery Horton, was widely regarded at NBS as the most successful experimenter in crystal growth. His work is exemplified by his large ammonium dihydrogen phosphate crystals and their habit modifications by ion additions, as well as by his purification of “ultrapure” reagent-grade benzene by melt recrystallization. He has left few direct traces of his influence in the literature except for two items, one on crystallography in chemical research [8], and another on a potential manufacturing application [9]. Torgesen’s expertise, however, was an important pillar in the creation of the International Conference on Crystal Growth, of its equivalent and active American Association for Crystal Growth, and, later, of the corresponding International Journal for Crystal Growth (North Holland). Starting with a Conference at Harvard University [10], many of these organizations have ever since been major vehicles for disseminating the rapidly expanding science and technology of crystal growth.

So numerous were the NBS contributions to such investigations that, in 1962, C. F. Yost of the Advanced Research Projects Agency (ARPA) of the U.S. Department of Defense agreed with I. C. Schoonover of NBS to fund jointly a 3 year program of unclassified research at NBS on crystal growth and characterization. At that time it had become clear that progress in science and technology often depended on breaking down barriers to cooperation between specialists in different disciplines or organizational divisions. Thus, Schoonover was anxious to experiment with “matrix management” across lines of organizational responsibilities without loss of the established NBS management structure. ARPA agreed to give the NBS program manager complete freedom of choice of tasks; while Schoonover gave instructions for many innovative tasks to be so supported, each with very little “seed” money. An important selection criterion was that each task had to be strongly supported also within the division from which it was proposed. In the event, in excess of a hundred separate tasks were so funded from 11—that is most—NBS research divisions. It is clearly impossible to describe here the individual summaries given in Refs. [11–14]. By a glance at this record one cannot but notice how many of these studies continued self-supported by divisions long beyond the time limit of this ARPA program and extended into contributions mentioned in Refs. [1] and [2] or in other papers in this Special Issue. They were intertwined with core research activities of very many groups at NBS that did not describe crystallography as their primary purpose. Yet, their favored research dealt with some of the basic problems of growing and characterizing crystals.

From among the many ARPA studies, let me first single out research by Hans Frederikse’s group on semiconductors based on *d*-electrons (as opposed to the more usual *s*-*p* electron conduction). They investigated the band structure of reduced strontium titanate (0.1 % to 0.01 % oxygen deficiency). It is cubic (perovskite structure) down to liquid helium temperatures. From knowledge of the density of states [15] and calculations of the energy band structure [16], they were able to prove theoretically and to confirm experimentally that this semiconductor becomes a superconductor, albeit at only 0.3 K, too low for this specific compound to have practical applications. It was work ahead of its time!

## 4. The Perfection of Crystals

Physical properties of crystals can be measured for characterizing the chemical and physical perfection of crystals. Among these properties the most important has probably been that of density. Its impact is described in Ref. [1] (see p. 193ff), but there the subjects are so condensed that it seems appropriate to emphasize here the cardinal role played by density standards and measuring methods developed at NBS. Combined with relative atomic-mass measurements they led directly to what remains one of the principal routes to the interconnected fundamental constants of physics. For the past 40 years, NBS/NIST has excelled in isotopic composition measurements which, with the highly accurately known isotope mass values, lead to the mean relative atomic mass values of polynuclidic elements in their characteristic terrestrial compositions. X-ray spacings in crystals, another great NBS specialty, and density of crystals of known chemical and isotopic composition yield the Avogadro constant by the XRCD (X-Ray Crystal Density) method. Its latest international evaluation has recently been published [17]. For stoichiometrically well characterized crystals the mean atomic or molecular mass values could be determined from the density and crystal cell volume alone without measuring isotopic composition [18]. In practice, however, to this date very few crystals have been of adequate physical and chemical perfection for this method to impact current knowledge embedded in the biennially revised tables of the relative atomic mass values of the elements [19]. Uniquely pure and dislocation-free silicon crystals are important exceptions that derive from the intense interest of the electronic industry. These crystals have been used for the previously mentioned measurement of the mean atomic mass of natural silicon and the Avogadro constant [17].

Earlier work on crystal perfection had to rely on external habit, some optical properties, and angular broadening of x-ray diffraction maxima. The last of these methods was exploited successfully at NBS especially in work on cements and fiber-forming polymers as described in other articles of this series, as seen also from Refs. [11–14]. The almost universal substructure of most crystals is characterized by mosaics, with slight relative misalignment. It permits the interpretation of diffraction intensities, by use of the kinematic theory, in terms of ionic, atomic, and molecular structure. During most of the century, this was by far the most important output of crystallographic studies. Those aspects, however, are not covered in this article, but NBS contributions on this topic are well represented elsewhere in this Special Issue.

In previously quoted Refs. [11–14] the interest in Laue’s original dynamical theory of x-ray diffraction came back into prominence, first by methods of x-ray imaging and also by exploiting anomalous x-ray transmission by highly perfect crystals. Deslattes and colleagues at NBS succeeded in simultaneously comparing x-ray transmission spacing periods with light interferometry on crystals moved smoothly on an atomic scale [1]. Equivalent procedures were used in the x-ray spacing measurements for silicon crystals for the XRCD method deriving the Avogadro constant [17].

## 5. The Characterization of Imperfections in Crystals

By mid-century, it became very clear that many of the most interesting properties of crystals were not those associated with the physical, chemical, and structural properties of the “ideally perfect” crystal, but were caused by characteristic crystal imperfections. It is not surprising that of the NBS projects described in Refs. [11–14], more than half deal with inclusions, isomorphous replacements, twinning, stacking faults, vacancies, other point defects, and, of course, dislocations.

In 1956, the NBS Free-Radical Program of coordinated unclassified research projects was established at NBS. This 3-year program was fully funded by the Department of the Army. The NBS program manager again had freedom of project choice. He did not have to seek, but often received, NBS division support. The basic ideas came from Herbert Broida and Arnold Bass, who had shown that small molecular fragments with an odd number of extranuclear electrons (i.e., free radicals) could be trapped in solid matrices at the melting point of helium [20]. With earlier experience in Britain of recording diffracted x-rays from specimens at high and low temperatures, I came to NBS to join Floyd Mauer and Leonard Bolz to build a liquid-helium Dewar for observing such solids with free radicals by x-ray powder diffraction and to examine their highly exothermic transformations on warming. The results were described in Chaps. 5 and 9 of Ref. [20]. The detection of free-radical H*, the potentially most interesting free radical, or N* within the crystal structures was neither expected, nor identified in substitutional or interstitial sites. The experiments with the free radicals did, however, reveal significant new details of low temperature phases including the polymorphic structures of water and ammonia. The significance to terrestrial upper atmospheric reactions of water and to possible ammonia seas of some other planet came under discussion. The current views on ice reactions in the cosmos were not foreseen [21].

A series of papers with Jack Watchman, such as [22], had the purpose to explore, by application of group theory, the symmetry of possible paths by which point defects could move around a special crystal site or with freedom in 1, 2, or 3 dimensions of a given crystal structure on overcoming distinct energy barriers. The observation of corresponding temperature change points would have been a challenge, but was not attempted. Deductions on internal friction were considered, but the simple derivation of the subgroup of a crystal’s symmetry by union with the symmetry of an outside mechanical stress or field was a simple output of this work [23].

Theoretical work on point defects and dislocations has produced an enormous literature to which NBS/NIST has contributed significantly. Justice is not done to that by here citing just two examples. Using a relaxation mode analysis, A. D. Franklin investigated the step-wise diffusion of atoms or ions into crystals [24] and recently, R. Thomson, working with L. E. Levine of Washington State University, has succeeded in deriving the effect of screw dislocations on Bragg x-ray scattering [25].

## Footnotes

1 Readers will recognize these and related contemporary NBS publications as essential groundwork for this year’s Chemistry Nobel Prizes awarded to W. S. Knowles, R. Noyori, and K. B. Sharpless for their chiral syntheses.
